# Inhibition of overexpressed Kv3.4 augments HPV in endotoxemic mice

**DOI:** 10.1186/s12890-020-01278-5

**Published:** 2020-10-08

**Authors:** Maurizio Turzo, Karin Metzger, Felix Lasitschka, Markus A. Weigand, Cornelius J. Busch

**Affiliations:** 1 Department of Anesthesiology, Heidelberg University Hospital, Im Neuenheimer Feld 110, 69120 Heidelberg, Germany; 2 Institute of Pathology, Heidelberg University Hospital, Heidelberg, Germany

**Keywords:** Hypoxic pulmonary vasoconstriction, Kv3.4, BDS-I, Mouse lung

## Abstract

**Background:**

Hypoxic pulmonary vasoconstriction (HPV) is a reaction of the pulmonary vasculature upon hypoxia, diverting blood flow into ventilated areas to preserve oxygenation. It is impaired in endotoxemia or ARDS. Voltage gated potassium channels have been shown to play a key role in the regulation of HPV. The aim of the study was to identify a voltage gated potassium channel involved in dysregulated HPV during endotoxemia.

**Methods:**

Lungs of male C57BL/6 mice with and without endotoxemia (*n* = 6 ea. group) were analyzed for Kv3.4 gene and protein expression. HPV was examined in isolated perfused lungs of mice with and without endotoxemia and with and without selective Kv3.4 blocker BDS-I (*n* = 7 ea. group). Pulmonary artery pressure (PAP) and pressure-flow curves were measured during normoxic (FiO_2_ 0.21) and hypoxic (FiO_2_ 0.01) ventilation. HPV was quantified as the increase in perfusion pressure in response to hypoxia in percent of baseline perfusion pressure (ΔPAP) in the presence and absence of BDS-I.

**Results:**

Kv3.4 gene (3.2 ± 0.5-fold, *p* < 0.05) and protein (1.5 ± 0.1-fold *p* < 0.05) expression levels were increased in endotoxemic mouse lungs. Endotoxemia reduced HPV (∆PAP control: 121.2 ± 8.7% vs. LPS 19.5 ± 8.0%, means ± SEM) while inhibition of Kv3.4 with 50 nM BDS-I augmented HPV in endotoxemic but not in control lungs (∆PAP control BDS-I: 116.6 ± 16.0% vs. LPS BDS-I 84.4 ± 18.2%, means ± SEM).

**Conclusions:**

Kv3.4 gene and protein expressions are increased in endotoxemic mouse lungs. Selective inhibition of Kv3.4 augments HPV in lungs of endotoxemic mice, but not in lungs of control mice.

## Background

Hypoxic pulmonary vasoconstriction (HPV) regulates pulmonary blood flow to match ventilation and optimize oxygenation [1]. HPV is diminished in humans and animals with pneumonia, sepsis or ARDS [2–4]. This accounts for an increase of intrapulmonary shunt with subsequent hypoxia [1]. During endotoxemia, a number of vasoactive mediators including nitric oxide (NO) and arachidonic acid metabolites modulate HPV, but the exact mechanisms mediating HPV remain not entirely understood [5].

Key regulators in HPV are voltage gated potassium channels, which also have been shown to regulate HPV in endotoxemia [4]. Nonspecific inhibition of voltage gated potassium channels with 4-AP increased HPV in an endotoxemic mouse model [4]. None of the analyzed potassium channels (Kv1.2, Kv1.5, Kv2.1, Kv3.1) in this study showed an increased expression, in fact, Kv1.2 was downregulated. So far there are 18 further voltage gated potassium channels reported to be expressed on RNA level in the pulmonary circulation [6]. Among others, Kv3.4, Kv6.3 and Kv9.3 have been described as oxygen sensitive or as regulative subunits for Kv2.1 [1, 6–9].

Kv3 channels assemble in mammals from subunits that are coded by four genes, named KCNC1–4 (Kv3.1–Kv3.4 subunits). Homomeric Kv3.1 and Kv3.2 channels, in heterologous expression systems, produce sustained currents, while Kv3.3 and Kv3.4 produce currents respectively with a slow and fast rate of inactivation [10]. Activation of Kv3-channels increases efflux of K^+^, resulting in increased closure of Ca^2^^+^ channels, decreased Ca^2^ ^+^ −influx and reduced intracellular Ca^2+^, which in turn leads to smooth muscle relaxation.

In humans, expression of Kv3.4 has been shown in pulmonary [3] and uterine smooth muscle cells, as well as in Purkinje cells [11, 12]. Besides vascular tone of pulmonary and splanchnic arteries, Kv3.4 is involved in the control of the cell cycle and proliferation of cells [12].

The aim of the study was to evaluate the role of Kv3.4, Kv6.3 and Kv9.3 in endotoxemic mouse lungs. We report that Kv3.4 gene as well as protein expressions were induced in endotoxemic mouse lungs. Gene expressions of Kv6.3 and Kv9.3 were unchanged. Immunohistochemistry showed a Kv3.4 immunoreactive protein in smooth muscle cells of mouse pulmonary arteries. HPV was reduced in lungs of endotoxemic mice. Inhibition of Kv3.4 with the specific blocker BDS-I increased HPV in isolated perfused endotoxemic mouse lungs.

## Methods

All animal experiments were conducted under protocols reviewed and approved by the Subcommittee on Research Animal Care of the University of Heidelberg (Regierungspräsidium Karlsruhe, Germany, AZ 35–9185.81/G-27/11) and handling of the animals was in accordance with the European Community guidelines. The study was performed with 51 male C57BL/6 mice (body weight 21.2 ± 1.0 g), animals were obtained from Charles River GmbH, Sulzfeld, Germany.

### Experimental groups

Mice for lung perfusion received endotoxin (LPS i.p. injection 0.5 ml; E.coli 0111:B4, 35 mg/kg bw; Sigma-Aldrich, St. Louis, MO, USA) 18 h before isolated lung perfusion experiments (LPS groups). During lung perfusion, BDS-I (Sigma-Aldrich) dissolved in Hanks’ balanced salt solution (Life Technologies, Paisley, UK) was added to give a final concentration in the perfusate of 50 nM (*n* = 7 animals in each group). Animals injected with 0.5 ml 0.9% NaCl solution served as controls (*n* = 7 animals in each group). For analysis of gene- and protein expression, as well as immunoenzyme staining, extra animals received an i.p. injection of endotoxin (*n* = 10) or 0.9% NaCl solution (*n* = 10).

### Isolated, perfused, and ventilated mouse lung model

Receiving pentobarbital sodium (300 mg/kg body weight i.p., Merial, Hallbergmoos, Germany), mice were sacrificed and lungs were explanted and buffer perfused as previously described (4). An in-line flow probe was used to adjust perfusate flow (Transonic Systems, Ithaca, NY, USA). Hanks’ balanced salt solution (Life Technologies) supplemented with bovine serum albumin (5%; Serva, Heidelberg, Germany) and dextran (5%; Sigma-Aldrich) to prevent pulmonary edema was used as perfusate as previously described [4]. In order to inhibit endogenous nitric oxide and prostaglandin synthesis, the nonselective nitric oxide synthase inhibitor L-NAME (1 mM L-arginine methyl ester; Sigma-Aldrich) and Indomethacin (30 mM; Sigma-Aldrich) were added to the perfusate. The perfusate pH was adjusted to 7.34–7.43 with sodium bicarbonate. Lungs were included in the study if they had a white homogenous appearance without signs of hemostasis or atelectasis, showing a stable perfusion pressure less than 10 mmHg during the second 5 min of the initial 10 min baseline perfusion period. Using these two criteria, three animals were excluded. Pulmonary artery pressure (PAP) and left atrial (LA) pressure were measured via 0.9% NaCl solution-filled membrane pressure transducers connected to a side port of the inflow and outflow cannulae. Pressure transducers were connected to a biomedical amplifier (4 Channel Bridge Amplifier TBM4M, World Precision Instruments, Berlin, Germany), and data were recorded at 150 Hz on a personal computer using an analog-to-digital interface with a data acquisition system (DI-220; Dataq Instruments, Akron, OH, USA). Before each experiment, the system was calibrated. HPV responsiveness (ΔPAP) was quantified as the difference between basal pulmonary arterial pressure (PAP) and PAP at the end of 6 min hypoxic ventilation (FiO_2_ 0.01).

### Lung wet/dry weight ratio

Both lungs of the studied animals (excluding their hilar structures) were excised and immediately weighed at the end of the experiment, dried in an oven at 100 °C overnight, and then re-weighted. Lung wet/dry weight ratios were calculated by dividing the wet weight by the dry weight as described previously [13].

### Semiquantitative RT-PCR

Mice were sacrificed after 18 h of LPS exposure (LPS; E.coli 0111:B4, 35 mg/kg bw i.p.; Sigma-Aldrich) with a lethal injection of pentobarbital sodium (Merial, 300 mg/kg bw i.p., *n* = 6). With 0.9% NaCl solution injected mice served as controls (*n* = 6). Lungs were exposed via median sternotomy and heparin (10 U) was injected into the right ventricle. Lungs were perfused with iced 0.9% NaCl solution for 1 min at 50 ml*kg-1*min-1 flow, dissected (excluding hilar structures), quick-frozen, and stored at − 80 °C.

RNA was isolated using the Trizol reagent (Invitrogen, Thermo Fisher Scientific, Waltham, MA, USA), and cDNA was generated with MMLV reverse transcriptase (Promega, Madison, WI, USA) and random primers (Promega). Quantitative RT-PCR was performed with the ABI Prism 7000 Sequence Detection System (Applied Biosystems, Foster City, CA, USA), using specific primers (see Table 1) and SYBR®Green PCR Master Mix (Applied Biosystems). To verify the presence of a single amplification product in the absence of DNA contamination, postamplification dissociation curves were performed. Changes of expression of the gene of interest were determined using the ∆∆Ct method with normalization to 18S ribosomal RNA.

**Table 1:** primer

	Forward primer	Reverse primer
Kv3.4	TTGACCGAAACGTGACGGAG	TGTAGGTAAGAATGGGCTCTGT
Kv6.3	GGGCACTACGCATCCTCTAC	CACAGCCAGGAAGAGCATCA
Kv9.3	GGACTCGGCGACCCGT	CCATCACATGCAGCTTCCCT
18S	TCAAGAACGAAAGTCGGAGG	GGACATCTAAGGGCATCAC

### Immunoblotting

Immunoblots were performed with tissue of mouse lungs to assess protein levels of Kv3.4 as well as the housekeeping protein GAPDH (glycerinaldehyd-3-phosphat-dehydrogenase). Samples of mouse lungs with and without endotoxemia (*n* = 6) were homogenized at 4 °C in RIPA buffer (50 mmol/L TRIS-HCL pH 7.5, 150 mmol/L NaCl, 1% Triton X-100, 0.1% SDS and protease inhibitor mix (Roche Diagnostics GmbH, Mannheim, Germany)) and centrifuged at 10,000 g, 4 °C for 10 min. Supernatant protein was subjected to electrophoresis, transferred to a PVDF membrane, and probed with rabbit anti-Kv3.4 (1:1000, Sigma-Aldrich) and mouse anti-GAPDH antibodies (1:10000, Merck, Darmstadt, Germany). As secondary antibodies served IRDye® 800CW goat anti-rabbit IgG (1:10000) and IRDye® 680RD goat anti-mouse (1:10000, IRDye®, LI-COR Biosciences, Lincoln, NE, USA). Proteins were visualized with a LICOR infrared imager (Odyssey, LI-COR Biosciences), quantitative densitometric analysis was performed by applying Odyssey version 1.2 infrared imaging software and signals were normalized to GAPDH.

### Immunoenzyme staining

Lungs of mice with and without endotoxemia were fixed in paraformaldehyde (*n* = 4 each group). After embedding in paraffin, 2 μm sections were cut using an automatic rotary microtome (MICROM HM 355, Thermo Fisher Scientific). Immunoenzyme stainings were performed using standard avidin-biotin anti-alkaline phosphatase technique (Vector Laboratories, Burlingame, CA, USA) according to the manufacturer’s instructions. Tris-buffered saline supplemented with 0.2% bovine serum albumin (Biotrend, Cologne, Germany) was used as buffer. Primary antibody dilutions of rabbit anti-Kv3.4, 1/50 (anti-KCNC4, SAB2101205, Sigma-Aldrich) and an isotype- and concentration-matched rabbit control Ig (Dianova, Hamburg, Germany) were prepared in this buffer and incubated for 1 h at room temperature. A biotinylated donkey anti-rabbit IgG Ab, 1/100 (Amersham™, GE Healthcare UK), was used as a secondary reagent (30 min at room temperature). Naphthol AS-biphosphate (Sigma-Aldrich) with New-fuchsin (Merck, Darmstadt, Germany) was used as the substrate for alkaline phosphatase. Slides were counterstained with hematoxylin (Sigma-Aldrich). Magenta positive staining (corresponding to Kv3.4 positive staining) was defined as the area of interest and was quantified (BX63 Upright Microscope; cellSens Dimension 1.17 software, Olympus, Tokyo, Japan).

### Statistical analysis

Data are reported as mean ± SEM. After approving the assumption of normality and equal variance across groups, differences were assessed using ANOVA followed by an appropriate post hoc comparison test. Groups were compared by a two-way ANOVA statistical test. When significant differences were detected by ANOVA, a post hoc least difference test for planned comparisons was used (SPSS 24, IBM, Armonk, USA). Statistical significance was assumed at a *p* value of less than 0.05.

## Results

### Endotoxemia increases pulmonary KCNC4 gene expression

**Fig. 1:**
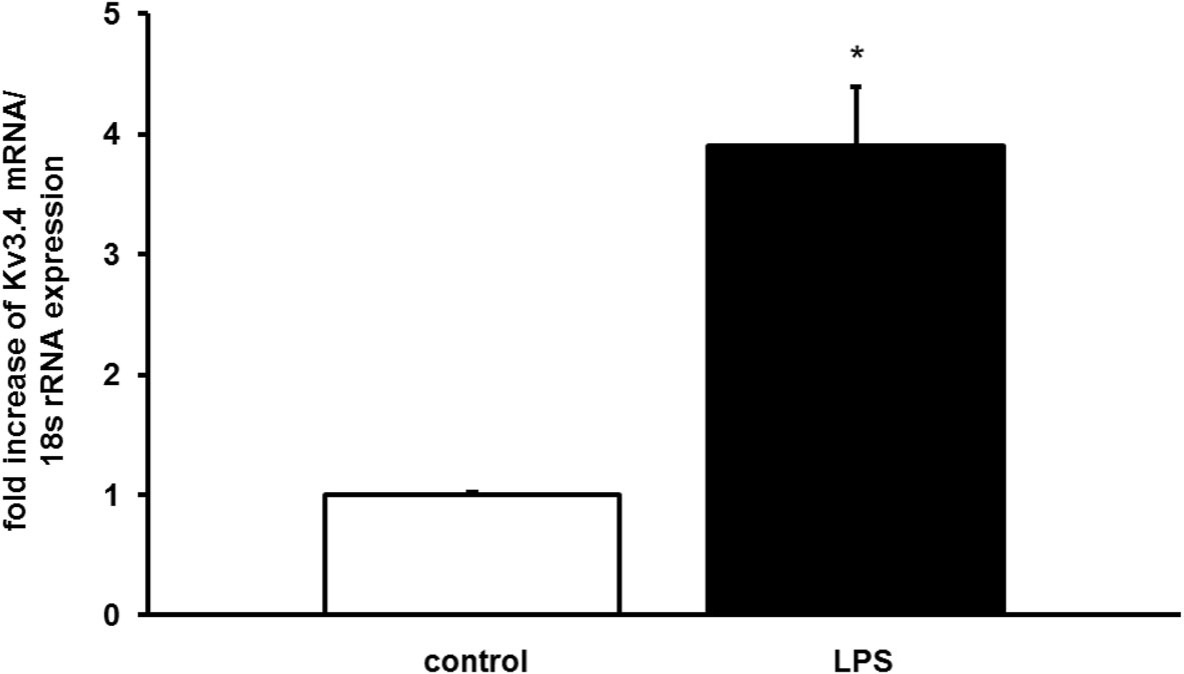
Kv3.4 gene expression is induced in mouse lungs after 18 h of endotoxemia. RNA was extracted from mice with and without LPS (rRNA: ribosomal RNA, representative QPCR, *n* = 6, means ± SEM, *P < 0.05 vs. control)

Extracts of total lung tissue from mice with LPS i.p. showed an increase of Kv3.4 gene expression (3.9 ± 0.5-fold, *n* = 6, mean ± SEM, *p* < 0.05) (Fig. 1) in comparison to 0.9% NaCl solution treated controls (1.0 ± 0.2-fold, normalized on 1, *n* = 6, means ± SEM). Gene expression levels of Kv6.3 and 9.3 were unaltered in LPS treated animals (Kv6.3: 1.4 ± 0.7; Kv9.3: 1.2 ± 0.6, *n* = 6, means ± SEM).

These results demonstrate that LPS increases mRNA expression of the voltage gated potassium channel Kv3.4.

### Endotoxemia increases pulmonary Kv3.4 protein expression

**Fig. 2:**
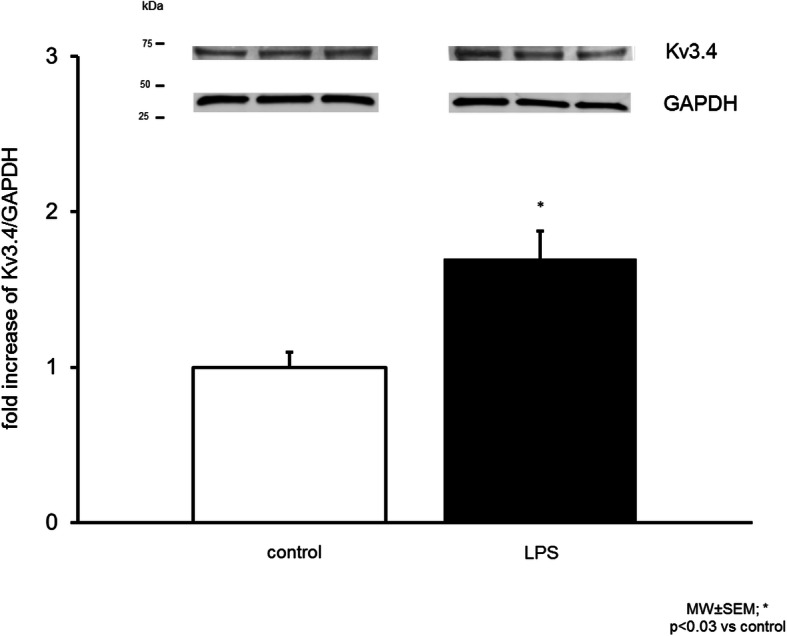
Expression of a Kv3.4 immunoreactive protein was increased in endotoxemic mouse lungs. Densitometric measurement of Kv3.4 bands (Representative immunoblot, *n* = 6, normalized to GAPDH, control normalized on 1, means ± SEM, *p < 0.05)

In order to measure if increased Kv3.4 gene expression is followed by increased protein expression, tissue of mouse lungs was extracted after 18 h of endotoxemia, 0.9% NaCl solution injected animals served as controls. Kv3.4 immunoreactive protein was increased in whole lung extracts in the LPS group (control 1.0 ± 0.2 vs. LPS 1.5 ± 0.1-fold; *n* = 6, *p* < 0.05, means ± SEM, Fig. 2).

These results show that LPS induces an increase of Kv3.4 protein expression in whole mouse lung extracts.

### Pulmonary expression of Kv3.4

**Fig. 3:**
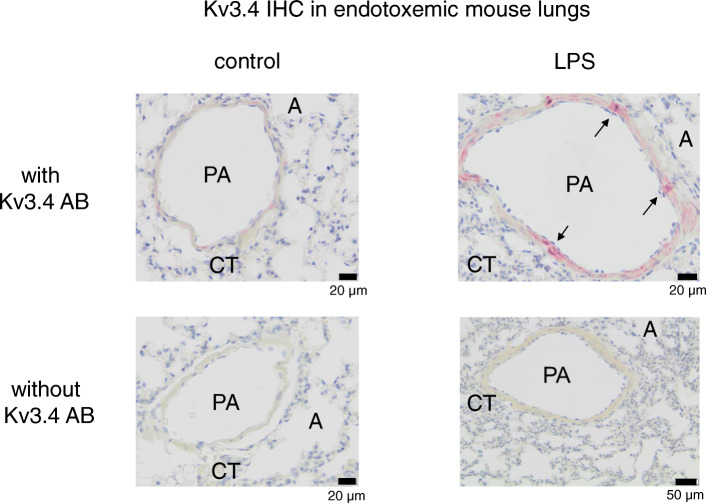
Kv3.4 immunoreactivity was measured in lungs of control mice (left) and lungs of mice after 18 h of endotoxemia (right). Immunoenzyme stainings were performed on paraffin embedded sections using rabbit anti-Kv3.4 1:50 (Sigma-Aldrich). Sections with an isotype- and concentration-matched rabbit control Ig served as negative controls (lower images). Smooth muscle cells of small pulmonary arteries show positive staining in control as well as endotoxemic animals (arrows). PA: pulmonary artery; A: alveole; CT: connective tissue; AB: antibody

In order to locate elevated Kv3.4 protein expression in mouse lungs, lungs (*n* = 4) with and without 18 h of endotoxemia where fixed in paraformaldehyde, embedded in paraffin and stained with Kv3.4 specific antibodies (Fig. 3). Positive staining of Kv3.4 immunoreactive protein was found in smooth muscle cells of small pulmonary arteries. Staining was only found in lung vessels. Magenta positive staining (corresponding to Kv3.4 immunoreactive protein) was measured in the area of pulmonary arteries and correlated to 3.5 ± 1.1% of the surface in controls vs. LPS 14.2 ± 3.0% (normalized on 1 control: 1.0 ± 0.3 vs. LPS 4.0 ± 0.9; *p < 0.0001, means ± SEM).

These results show that increased Kv3.4 expression in endotoxemia can be attributed to smooth muscle cells in pulmonary arteries.

### Pulmonary vascular response to hypoxic ventilation in control mice as well as endotoxemic mice

**Fig. 4:**
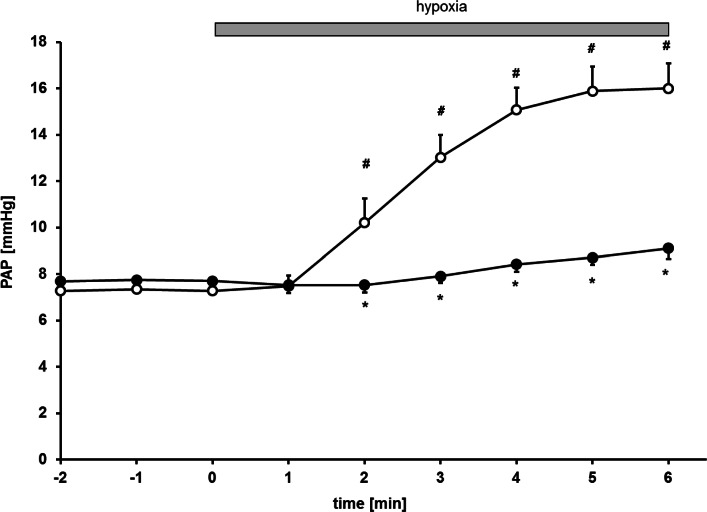
Time course of pulmonary artery pressure (PAP) under normoxic baseline (− 2–0 min) and during hypoxic ventilation (1–6 min; hypoxia) in lungs of LPS-pretreated (●) and untreated control mice (○). (*p < 0.05 corresponding control, #*p* < 0.05 vs. normoxia baseline, *n* = 7 per group, mean ± SEM)

Hypoxic ventilation of lungs of control mice caused an increase of PAP from 7.3 ± 0.5 to 16.0 ± 1.1 mmHg (∆PAP: 8.7 mmHg, Fig. 4). These results demonstrate that ventilation of an isolated perfused control mouse lung with hypoxic gas increases PAP.

**Fig. 5:**
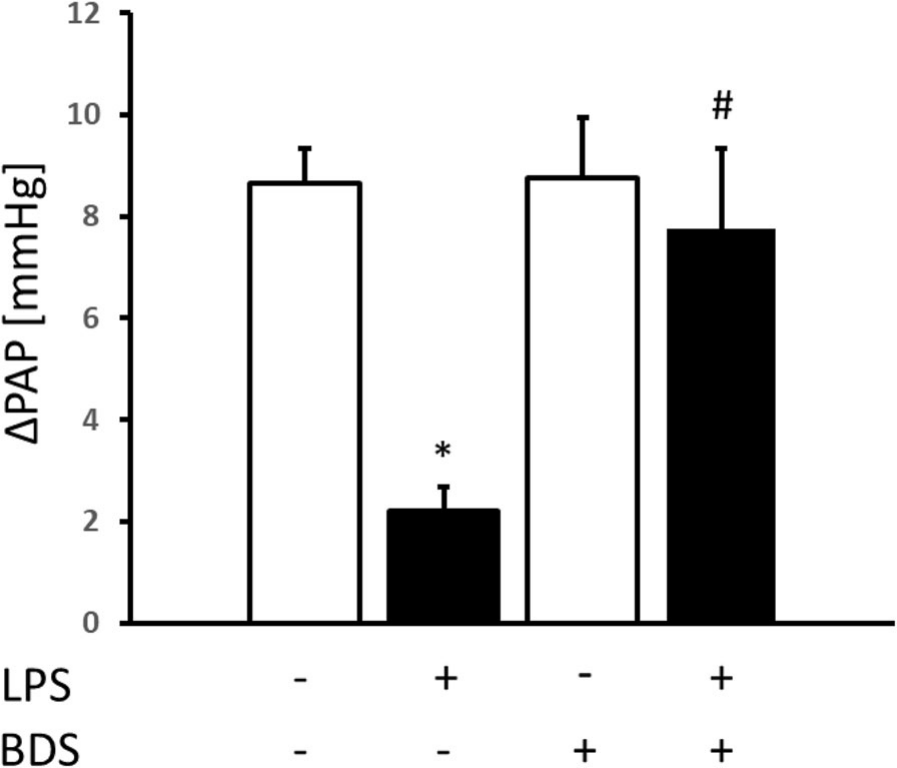
Effects of lung perfusion with and without 50 nM of the Kv3.4 inhibitor BDS-I on hypoxia induced vasoconstriction expressed as increase mmHg of baseline perfusion pressure (ΔPAP) in lungs obtained from LPS-treated (■) and untreated control (□) mice (*p < 0.05 vs. control; # *p* < 0.05 versus LPS without BDS-I; *n* = 7 per group, means ± SEM)

Initial perfusion pressure (representing pulmonary artery pressure) under normoxic ventilation showed no difference between untreated and LPS-treated mice (control 7.3 ± 0.5 mmHg vs. LPS 7.7 ± 0.4 mmHg, Fig. 4). Pulmonary artery pressure increased upon hypoxic ventilation (FiO_2_ of 0.01) in lungs of control mice (Figs. 4 and 5; ∆PAP 8.7 mmHg), while lungs of endotoxemic mice showed an attenuated increase after 6 min of hypoxic ventilation (Figs. 4 and 5; ∆PAP 2.2 mmHg).

Taken together, this data shows that hypoxia induces HPV in lungs of control mice and endotoxemia attenuates HPV.

### Inhibition of Kv3.4 with BDS-I increases HPV after LPS

In order to test the role of Kv3.4 in the pulmonary vasculature, lungs of mice with and without endotoxemia were perfused with and without 50 nM of the Kv3.4 specific inhibitor BDS-I. Perfusion of untreated control mice with 50 nM BDS-I did not affect baseline PAP under normoxia (control 7.3 ± 0.5 mmHg vs. BDS-I 7.8 ± 0.4 mmHg). Again, rise of PAP started within 2 min of hypoxia and reached its maximum within 6 min. Perfusion with the inhibitor BDS-I did not augment HPV in control mice (Fig. 5; control without BDS-I ∆PAP 8.7 ± 0.7 mmHg vs. control with BDS-I ∆PAP 8.8 ± 1.2 mmHg).

Blockade of Kv3.4 with BDS-I in lungs of endotoxemic mice did not alter baseline pressures (LPS 7.7 ± 0.4 mmHg vs. LPS/BDS-I 8.1 ± 0.2 mmHg). In contrast, specific inhibition of Kv3.4 in lungs of endotoxemic mice augmented HPV (∆PAP 7.8 ± 1.6 mmHg) compared to lungs perfused without inhibitor (∆PAP 2.2 ± 0.5 mmHg) (Fig. 5).

This shows that BDS-I does not alter HPV in control but augments HPV in endotoxemic mice.

## Discussion

This study investigated the role of the voltage-gated potassium channel Kv3.4 on HPV in endotoxemic mice. While lungs of control mice showed solid HPV, it was impaired in endotoxemia. Exposure of mice to LPS increased gene as well as protein expressions of Kv3.4 in lung tissue. This induced Kv3.4 expression was attributable to pulmonary artery smooth muscle cells. Pharmacological inhibition of Kv3.4 in LPS treated mice restored in part HPV, while it did not affect HPV in control animals.

In line with other studies, a strong HPV was found in buffer-perfused isolated mouse lungs of healthy animals [14, 15]. Consistent with previous experiments, a distinctive reduction of HPV was observed in lungs of endotoxemic mice [4, 16]. It has been shown that HPV is modulated by several vasoactive mediators including nitric oxide and arachidonic acid metabolites, but the precise mechanisms mediating HPV are still incompletely understood [15–19]. Increased activity of NOS2 [13] results in elevated sGC activity with subsequent induction of the effector proteins of sGC in smooth muscle cells. This alters PKG and Ca^2+^ signaling CREB dependent in PASMC, which in turn leads to vasodilatation and reduced HPV. Moreover, expression of voltage gated potassium channels is regulated by CREB [20]. Further mechanisms altering HPV in endotoxemia include leukotrienes, which are induced and have been shown to reduce HPV in mice. Inhibition of 5-lipoxygenase (5-LO) \with MK886 in endotoxemic mice augmented HPV and endotoxemic 5-LO knock-out mice showed persistent HPV [21].

Another regulatory protein of HPV is PKC [22]. Inhibition of PKC reduced HPV in isolated blood perfused dog lungs while activation of PKC increased HPV [23]. Ahn et al. found a similar proinflammatory cytokine response in PKC-δ knock-out mice after intratracheal LPS administration when compared to wild type mice, but increased pulmonary neutrophil infiltration and perivascular edema [24]. This suggests that PKC-δ is not involved in the regulation of proinflammatory mediators, but plays a role in the control of vascular permeability and transmigration of neutrophils through the endothelium [24].

Potassium channels have been shown to play a key role in HPV [1]. Endotoxemia induced overexpression of one or more Kv channels in the pulmonary vascular bed contribute to loss of HPV [4]. Numerous voltage gated potassium channels, like Kv1.2, Kv1.5, Kv2.1, Kv3.1, Kv3.4, Kv6.3 as well as Kv9.3 have been reported to be oxygen sensitive and expressed in the pulmonary vasculature [1, 7, 25, 26]. Of these, gene expressions of Kv1.2, Kv1.5, Kv2.1 and Kv3.1 were found to be unchanged or decreased in extracts of endotoxemic whole mouse lungs [4]. Although it seems less likely that these channels are responsible for loss of HPV in endotoxemia, it cannot be ruled out that heterogenic expression of a channel within the course of a vessel is missed by QPCR of whole lung tissue [10]. Another possibility is an alternated function of oxygen sensitive potassium channels, like it has been reported for Kv2.1/Kv9.3 [27]. In Xenopus oocytes and transfected COS cells, coexpression of Kv2.1/Kv9.3 consistently increased channel current amplitude and shifted steady-state activation towards negative values. Since Kv9.3 gene expression was unaltered by LPS, this mechanism is unlikely responsible for loss of HPV in endotoxemia.

Presence of Kv6.3 was first described in the tissue of rat brains [28], but is also present in PASMC [6] and functions as a modulatory subunit for Kv2.1 [28]. Heterodimeric Kv2.1/Kv6.3 channels showed a reduced rate of deactivation compared to Kv2.1 channels alone [28]. Since Kv6.3 gene expression was unchanged in LPS lungs, alteration of HPV through Kv6.3 in endotoxemia is unlikely. Further oxygen sensitive channels expressed in the pulmonary vasculature include Kv4.3 and Kv7 [6, 29], which have not been investigated in these experiments.

Kv3.4 was not only present in pulmonary arteries of mice, but also detected in rat PASMC [8]. Kv3.4 is reported to be expressed in cultured human PASMC as well as in intact human main and small pulmonary arteries [6, 30]. Reduced Kv3.4 expression has been reported in cultured rat PASMC after 24 h of hypoxia, this effect was dependent on 15-LO [8]. We did not measure Kv3.4 expression after the brief hypoxia in our experimental setting, but it is unlikely that expression changed within or shortly after 10 min of hypoxia.

Kv3.4 channel activity was altered in vitro by phosphorylation of the N-terminus through PKC, converting Kv3.4 to a non-inactivating delayed rectifier type channel [30]. Since LPS induced PKC in vitro within 6 h in mouse microglia [31] and after 12 h in mouse lungs [32], it is possible that not only the number of Kv3.4 channels is increased by LPS, but also the amount of open channels.

The sea anemone venom BDS-I is a 43 amino acid peptide and described as selective Kv3.4 inhibitor, mostly inactive to other Kv3 subchannels [33]. In Xenopus oocyte expressed Kv channels, highest selectivity was described for Kv3.4 with an IC50 of 47 nM, some inhibitory effects were found for Kv1.2, Kv1.3 and Kv3.1 at a dose of 10 μM [33]. The K0.5 value for BDS-I in rat brain receptors was 12 nM [33]. Besides Kv3.4, BDS-I showed inhibiting properties for Kv3.1a and Kv3.2b at a dose of 500 nM (45 and 48%) [33]. Since a ten times lower concentration was used in the isolated perfused mouse lung, inhibition with 50 nM BDS-I most likely blocked predominantly Kv3.4.

Besides Kv3.4 channels, BDS-I also shows inhibitory properties to voltage-sensitive Na^+^-channels, especially Nav1.7 at a concentration of 500 nM. This voltage gated sodium channel can be found in the heart, skeletal muscle, placenta, pancreatic β-cells as well as the brain and sensory and sympathetic neurons of the peripheral nervous system [34]. Only the neuronal Nav-channels show relevant sensitivity to BDS-I [35]. Since Nav1.7 expression is not described in pulmonary arteries, it reduces the possibility that the effect by 50 nM BDS-I in the isolated perfused mouse lung are provoked via inhibition of Nav1.7.

In inactive smooth muscle cells, membrane potential is stabilized by open K-channels maintaining an outward flux of K^+^. This prevents voltage sensitive Ca^2+^-channels from opening, resulting in vasodilatation. However, if outward K^+^ flux is decreased through inhibition of Kv channels, membrane potential is shifted to more positive potential, which in turn opens more Ca^2+^-channels causing vasoconstriction induced by Ca^2+^-influx. A hypothetical mechanism by which HPV is increased in endotoxemia is the inhibition of overexpressed Kv3.4 that would augment HPV through depolarizing the membrane and increasing the open-state probability of Ca^2+^-channels.

An isolated, perfused mouse lung model was used to study pulmonary vasoreactivity in response to acute hypoxia in LPS exposed mice. Several limitations of this experimental setup have to be considered when extrapolating our data to mice in vivo or even to septic humans. The initial normoxic PAP showed no difference between untreated and LPS-treated mice. In contrast, patients with ARDS or animals with endotoxemia suffer from elevated PAP and vascular hyperreactivity [36]. This is most likely due to the model of the isolated perfused mouse lung that was used. Hanks’ balanced salt solution as perfusate without recirculation excludes thrombosis and perfusion with vasoconstrictive mediators. Furthermore a stable outflow pressure of 2 mmHg (equaling left atrial pressure) and avoiding atelectasis by explanting and expanding the lung also contribute to the missing increase of PAP in endotoxemia. The cyclooxygenase inhibitor indomethacin as well as the inhibitor of soluble guanylate cyclase, L-NAME, were added to the perfusate to generate a robust HPV [15]. Simultaneous inhibition of NO and prostaglandin production do not alter baseline pulmonary pressures in healthy isolated mouse lungs, but upon hypoxia [15]. An i.p. LPS model was used to induce inflammation. Although it does not reproduce the full clinical scale of sepsis in humans, it has advantages. It is better reproducible compared to cecal ligation and puncture or colon ascendens stent peritonitis models and has no need for a surgical intervention before starting an experiment [37, 38]. One of the disadvantages of the LPS model is the difficulty of transferability into human pathophysiology.

Almost 10% of the patients admitted to intensive care units due to severe sepsis develop ARDS [39]. In these patients, at least part of their hypoxemia can be attributed to loss of HPV in sepsis. Therapeutic pulmonary vasoconstriction for the treatment of hypoxemia due to ARDS has been tested before using vasoconstrictors such as norepinephrine, phenylephrine or almitrine in combination with inhaled nitric oxide, without clinical benefit [40–42]. So far there is still no data on the expression of potential target channels in septic patients with acute lung injury, but selective inhibition of upregulated voltage gated potassium channels in these patients might be a therapeutic tool to restore HPV and to prevent hypoxia.

## Conclusion

In summary, endotoxemia induces in mouse lungs expression of Kv3.4. This increased expression is located in pulmonary arteries. The Kv3.4 inhibitor BDS-I acutely increased HPV in endotoxemic mice but not in controls. Selective inhibition of in endotoxemia overexpressed voltage gated potassium channels are potential targets to increase HPV in gram negative induced respiratory failure.

## Data Availability

The datasets used and analysed during the current study are available from the corresponding author on request.

## Abbreviations

A: alveole
AB: antibody
ARDS: acute respiratory distress syndrome
BDS: Blood depressing substance I
cDNA: complementary deoxyribonucleic acid
COS: monkey kidney cell line carrying the simian virus 40CT
CREB: cAMP response element binding protein
GAPDH: glyceraldehyde 3-phosphate dehydrogenase
HPV: hypoxic pulmonary vasoconstriction
i.p.: intraperitoneal
KCNC: gene potassium voltage-gated channel
Kv: voltage gated potassium channel
L-NAME: L-arginine methyl ester
MMLV: Moloney Murine Leukemia Virus
mRNA: messenger ribonucleic acid
NaCl: sodium chloride
NaV: voltage-gated sodium channel
NO: nitric oxide
NOS: nitric oxide synthetase
PA: pulmonary artery
PAP: pulmonary artery pressure
PASMC: pulmonary artery smooth muscle cell
pH: potential for hydrogen
PKC: protein kinase C
PKG: protein kinase G
PVDF: polyvinylidene difluoride
QPCR: quantitative polymerase
RIPA: radioimmunoprecipitation assay buffer
RNA: ribonucleic acid
rRNA: ribosomal ribonucleic acid
SEM: standard of error mean
sGC: soluble guanylate cyclase
5-LO: 5- lipoxygenase

## References

[1] Moudgil R, Michelakis ED, Archer SL (2005). Hypoxic pulmonary vasoconstriction. J Appl Physiol.

[2] Reeves JT, Grover RF (1974). Blockade of acute hypoxic pulmonary hypertension by endotoxin. J Appl Physiol.

[3] Marshall BE, Hanson CW, Frasch F, Marshall C (1994). Role of hypoxic pulmonary vasoconstriction in pulmonary gas exchange and blood flow distribution. 2. Pathophysiology. Intensive Care Med.

[4] Spöhr F, Busch CJ, Reich C, Motsch J, Gebhard MM, Kuebler WM, Bloch KD, Weimann J (2007). 4-Aminopyridine restores impaired hypoxic pulmonary vasoconstriction in endotoxemic mice. Anesthesiology..

[5] Petersen B, Busch CJ, Schleifer G, Schaack D, Lasitschka F, Bloch KD, Bloch DB, Ichinose F (2019). Arginase impairs hypoxic pulmonary vasoconstriction in murine endotoxemia. Respir Res.

[6] Firth AL, Remillard CV, Platoshyn O, Fantozzi I, Ko EA, Yuan JX (2011). Functional ion channels in human pulmonary artery smooth muscle cells: voltage-dependent cation channels. Pulm Circ.

[7] Davies AR, Kozlowski RZ (2001). Kv channel subunit expression in rat pulmonary arteries. Lung..

[8] Guo L, Tang X, Tian H, Liu Y, Wang Z, Wu H, Wang J, Guo S, Zhu D (2008). Subacute hypoxia suppresses Kv3.4 channel expression and whole-cell K+ currents through endogenous 15-hydroxyeicosatetraenoic acid in pulmonary arterial smooth muscle cells. Eur J Pharmacol.

[9] Park WS, Firth AL, Han J, Ko EA (2010). Patho-, physiological roles of voltage-dependent K+ channels in pulmonary arterial smooth muscle cells. J Smooth Muscle Res.

[10] Rudy B, McBain CJ (2001). Kv3 channels: voltage-gated K+ channels designed for high-frequency repetitive firing. Trends Neurosci.

[11] Sacco T, De Luca A, Tempia F (2006). Properties and expression of Kv3 channels in cerebellar Purkinje cells. Mol Cell Neurosci.

[12] Miguel-Velado E, Pérez-Carretero FD, Colinas O, Cidad P, Heras M, López-López JR, Pérez-García MT (2010). Cell cycle-dependent expression of Kv3.4 channels modulates proliferation of human uterine artery smooth muscle cells. Cardiovasc Res.

[13] Weimann J, Bloch KD, Takata M, Steudel W, Zapol WM (1999). Congenital NOS2 deficiency protects mice from LPS-induced hyporesponsiveness to inhaled nitric oxide. Anesthesiology..

[14] Turzo M, Vaith J, Lasitschka F, Weigand MA, Busch CJ (2018). Role of ATP-sensitive potassium channels on hypoxic pulmonary vasoconstriction in endotoxemia. Respir Res.

[15] Weissmann N, Akkayagil E, Quanz K, Schermuly RT, Ghofrani HA, Fink L, Hänze J, Rose F, Seeger W, Grimminger F (2004). Basic features of hypoxic pulmonary vasoconstriction in mice. Respir Physiol Neurobiol.

[16] Jahn N, Lamberts RR, Busch CJ, Voelker MT, Busch T, Koel-Simmelink MJ, Teunissen CE, Oswald DD, Loer SA, Kaisers UX, Weimann J (2015). Inhaled carbon monoxide protects time-dependently from loss of hypoxic pulmonary vasoconstriction in endotoxemic mice. Respir Res.

[17] Sylvester JT, Shimoda LA, Aaronson PI, Ward JP (2012). Hypoxic pulmonary vasoconstriction. Physiol Rev.

[18] Ullrich R, Bloch KD, Ichinose F, Steudel W, Zapol WM (1999). Hypoxic pulmonary blood flow redistribution and arterial oxygenation in endotoxin-challenged NOS2-deficient mice. J Clin Invest.

[19] Ichinose F, Hataishi R, Wu JC, Kawai N, Rodrigues AC, Mallari C, Post JM, Parkinson JF, Picard MH, Bloch KD, Zapol WM (2003). A selective inducible NOS dimerization inhibitor prevents systemic, cardiac, and pulmonary hemodynamic dysfunction in endotoxemic mice. Am J Physiol Heart Circ Physiol.

[20] Mori Y, Matsubara H, Folco E, Siegel A, Koren G (1993). The transcription of a mammalian voltage-gated potassium channel is regulated by cAMP in a cell-specific manner. J Biol Chem.

[21] Ichinose F, Zapol WM, Sapirstein A, Ullrich R, Tager AM, Coggins K, Jones R, Bloch KD (2001). Attenuation of hypoxic pulmonary vasoconstriction by endotoxemia requires 5-lipoxygenase in mice. Circ Res..

[22] Weissmann N, Voswinckel R, Hardebusch T, Rosseau S, Ghofrani HA, Schermuly R, Seeger W, Grimminger F (1999). Evidence for a role of protein kinase C in hypoxic pulmonary vasoconstriction. Am J Phys.

[23] Barman SA (2001). Effect of protein kinase C inhibition on hypoxic pulmonary vasoconstriction. Am J Physiol Lung Cell Mol Physiol..

[24] Ahn JJ, Jung JP, Park SE, Lee M, Kwon B, Cho HR (2015). Involvement of protein kinase C-δ in vascular permeability in acute lung injury. Immune Netw.

[25] Hulme JT, Coppock EA, Felipe A, Martens JR, Tamkun MM (1999). Oxygen sensitivity of cloned voltage-gated K(+) channels expressed in the pulmonary vasculature. Circ Res.

[26] Sommer N, Dietrich A, Schermuly RT, Ghofrani HA, Gudermann T, Schulz R, Seeger W, Grimminger F, Weissmann N (2008). Regulation of hypoxic pulmonary vasoconstriction: basic mechanisms. Eur Respir J.

[27] Patel AJ, Lazdunski M, Honore E (1997). Kv2.1/Kv9.3, a novel ATP-dependent delayed-rectifier K-channel in oxygen-sensitive pulmonary artery myocytes. EMBO J.

[28] Sano Y, Mochizuki S, Miyake A, Kitada C, Inamura K, Yokoi H, Nozawa K, Matsushime H, Furuichi K (2002). Molecular cloning and characterization of Kv6.3, a novel modulatory subunit for voltage-gated K(+) channel Kv2.1. FEBS Lett.

[29] Sedivy V, Joshi S, Ghaly Y, Mizera R, Zaloudikova M, Brennan S, Novotna J, Herget J, Gurney AM (2015). Role of Kv7 channels in responses of the pulmonary circulation to hypoxia. Am J Physiol Lung Cell Mol Physiol.

[30] Iida H, Jo T, Iwasawa K, Morita T, Hikiji H, Takato T, Toyo-Oka T, Nagai R, Nakajima T (2005). Molecular and pharmacological characteristics of transient voltage-dependent K+ currents in cultured human pulmonary arterial smooth muscle cells. Br J Pharmacol.

[31] Covarrubias M, Wei A, Salkoff L, Vyas TB (1994). Elimination of rapid Potassium Channel inactivation by phosphorylation of the inactivation gate. Neuron..

[32] Fu SY, Xiong RP, Peng Y, Zhang ZH, Chen X, Zhao Y, Ning YL, Yang N, Zhou YG, Li P (2019). PKC mediates LPS-induced IL-1β expression and participates in the pro-inflammatory effect of A2AR under high glutamate concentrations in mouse microglia. Neurochem Res.

[33] Diochot S, Schweitz H, Béress L, Lazdunski M (1998). Sea anemone peptides with a specific blocking activity against the fast inactivating potassium channel Kv3.4. J Biol Chem.

[34] Yeung SY, Thompson D, Wang Z, Fedida D, Robertson B (2005). Modulation of Kv3 subfamily potassium currents by the sea anemone toxin BDS: significance for CNS and biophysical studies. J Neurosci.

[35] Saleh S, Yeung SY, Prestwich S, Pucovs.ky V, Greenwood I. Electrophysiological and molecular identification of voltage-gated sodium channels in murine vascular myocytes. J Physiol. 2005; 568: 155–169. 10.1113/jphysiol.2005.090951 PMC1474751 16020462

[36] Held HD, Uhlig S (2000). Mechanisms of endotoxin-induced airway and pulmonary vascular hyperreactivity in mice. Am J Respir Crit Care Med.

[37] Matute-Bello G, Frevert CW, Martin TR (2008). Animal models of acute lung injury. Am J Physiol Lung Cell Mol Physiol.

[38] Traeger T, Koerner P, Kessler W, Cziupka K, Diedrich S, Busemann A, Heidecke CD, Maier S (2010). Colon Ascendens stent peritonitis (CASP) - a standardized model for Polymicrobial abdominal Sepsis. J Vis Exp.

[39] Mikkelsen ME, Shah CV, Meyer NJ (2013). The epidemiology of acute respiratory distress syndrome in patients presenting to the emergency department with severe sepsis. Shock.

[40] Doering EB, Hanson CW, Reily DJ, Marshall C, Marshall BE (1997). Improvement in oxygenation by phenylephrine and nitric oxide in patients with adult respiratory distress syndrome. Anesthesiology.

[41] Mourgeon E, Puybasset L, Law-Koune JD, Lu Q, Abdennour L, Gallart L, Malassine P, Rao GU, Cluzel P, Bennani A, Coriat P, Rouby JJ (1997). Inhaled nitric oxide in acute respiratory distress syndrome with and without septic shock requiring norepinephrine administration: a dose-response study. Crit Care.

[42] Gallart L, Lu Q, Puybasset L, Umamaheswara RG, Coriat P, Rouby JJ (1998). Intravenous almitrine combined with inhaled nitric oxide for acute respiratory distress syndrome. The NO Almitrine study group. Am J Respir Crit Care Med.

